# Metabolite Mapping with Extended Brain Coverage Using a Fast Multisection MRSI Pulse Sequence and a Multichannel Coil

**DOI:** 10.1155/2012/247161

**Published:** 2012-02-20

**Authors:** Zhengchao Dong, Feng Liu, Alayar Kangarlu, Bradley S. Peterson

**Affiliations:** ^1^Department of Psychiatry, Columbia University College of Physicians & Surgeons, New York State Psychiatric Institute, New York, NY 10032, USA; ^2^Department of Psychiatry, Columbia University and New York State Psychiatric Institute, 1051 Riverside Drive, No. #74, New York, NY 10032, USA

## Abstract

Multisection magnetic resonance spectroscopic imaging is a widely used pulse sequence that has distinct advantages over other spectroscopic imaging sequences, such as dynamic shimming, large region-of-interest coverage within slices, and rapid data acquisition. It has limitations, however, in the number of slices that can be acquired in realistic scan times and information loss from spacing between slices. In this paper, we synergize the multi-section spectroscopic imaging pulse sequence with multichannel coil technology to overcome these limitations. These combined techniques now permit elimination of the gaps between slices and acquisition of a larger number of slices to realize the whole brain metabolite mapping without incurring the penalties of longer repetition times (and therefore longer acquisition times) or lower signal-to-noise ratios.

## 1. Introduction

The applications of proton magnetic resonance spectroscopic imaging (1H MRSI) of the brain can benefit from technical developments in pulse sequences and hardware advances to overcome various limitations of MRSI, including low SNR, long acquisition times, and lipid contamination. In pulse sequence development, 2D PRESS-MRSI [1] and 2D STEAM-MRSI [2] have been developed to address several of these limitations and are now widely used. By exciting only a small region of interest within the brain, both sequences permit reduction in the field of view (FOV), which in turn permits a reduction in the number of phase encoding steps and thereby reduces the scan time required to achieve a given spatial resolution [3]. These pulse sequences also reduce contamination of the metabolite signals by lipid signals from the scalp. These and other advantages have motivated the extension of PRESS- and STEAM-MRSI from conventional uses to 3D or multiple 2D applications, or to their combination with other techniques, such as echo-planar spectroscopic imaging [4] and spiral MRSI [5]. PRESS and STEAM MRSI, however, also have several disadvantages, such as relatively small brain coverage and scan times that are still long for human applications, especially when used in 3D or multiple 2D modes [6]. 

Another development in fast MRSI sequences has been multi-section MRSI [7]. This sequence features two advantages over other PRESS- or STEAM-based fast sequences. (1) Multiple slices are consecutively excited and sampled within one repetition time (*TR*), whereas lipid signals from subcutaneous fat are suppressed through the application of oblique saturation bands placed by the user on localizer images [3]. (2) Each slice is dynamically shimmed, and therefore better spectral quality can be achieved than with global shimming. In addition, multi-section MRSI also possesses the following features. (1) Full echoes, instead of free induction decays (FIDs), are acquired, so that magnitude spectra can be used without employing phase correction. Acquiring full echoes offers an additional advantage for the reconstruction of MRSI signals sampled using a multichannel receiver RF coil, in that the residual water signals can be used as sensitivity references when combining signals from the various channels of the coil [8]. (2) Spacing between slices avoids the signal cancellation caused by “crosstalk” between adjacent slices.

**Figure 1:**
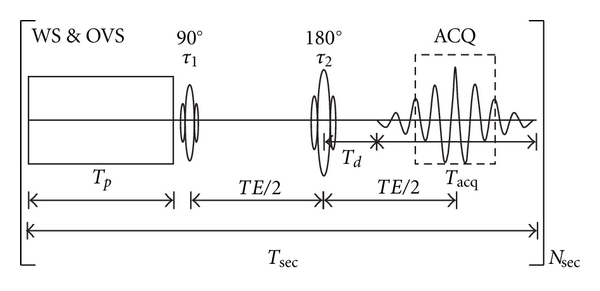
Pulse sequence diagram and timing for multi-section MRSI. The *T_p_* is the time for the preparation period, which includes water suppression and outer volume suppression; *τ*_1_ and *τ*_2_ are the durations of 90° and 180° pulses, respectively; *TE* is the spin echo time; *T_d_* is the time delay before data acquisition, which is used for slice selection in the *z*-direction and phase encoding in the *x*-*y* plane of the slice (not shown); *T*_acq_ is the acquisition time; *T*_sec_ is the time for one slice, and the total repetition time *TR* for *N*_sec_ slices is *TR* = *T*_sec_ × *N*_sec_. WS: water suppression; OVS: outer volume suppression; ACQ: acquisition.

The conventional implementation of multisection MRSI technique [7] also has attendant problems, however. The spacing required between slices fails to acquire information throughout the entire imaging volume, not only losing important information but also creating difficulties for the coregistration of high-resolution MRI images with the MRSI data, which is important for the segmentation of MRSI slices and the analysis of the MRSI data. Although the use of a 288 ms echo time (*TE*) is necessary to accommodate the full number of echoes for 512 data points at a sampling rate of 2000 Hz, it also lowers the SNR and requires a long *TR* of 2.3 seconds to sample phase-encoded signals from 4 slices. Because each *TR* is “full” (Figure 1), any further increase in the number of slices is possible only by linearly increasing the *TR*, which undermines the “fast” feature of the pulse sequence. The limited number of slices also limits the volume coverage, requiring an increase in slice thickness and a trade-off with compromising spatial resolution to cover a larger volume with the same 4 slices.

Hardware development, and the use of multichannel coils in particular, has benefitted the applications of 1H MRSI. The initial and conventional application of multichannel coils in MRS was to improve SNR [9–12], as the array of surface coils provides superior sensitivity compared with more conventional quadrature volume coils [10]. In recent years, parallel MRSI has employed multichannel coils to accelerate data acquisition [13]. The gains in SNR or the reduction in scan time, though valuable in their own right, can be further traded for other potential advantages, such as spatial resolution of MRSI [14].

We note that the limitations of multi-section MRSI are not caused by intrinsic disadvantages of the sequence, but rather are the consequence of technical limitations and compromises that can be addressed powerfully using multichannel coil technology. We hypothesize that the use of multichannel coil technology for multi-section spectroscopic imaging can eliminate the need for gaps between slices and permit an increase in the number of slices without resorting to longer acquisition times or lower spatial resolution for improved brain coverage. Therefore, our aim in this report is to synergize the multi-section spectroscopic imaging (SI) pulse sequence [7] with multichannel coil technology to realize whole brain metabolite mapping by removing the gaps between slices and increasing the number of slices without resorting to trading off longer *TR*s and therefore longer acquisition times or poorer spatial resolution for more brain coverage.

## 2. Methods

### 2.1. MRSI Sequence

The timing of the simplified RF pulse sequence for multi-section MRSI is schematically shown in Figure 1. *T_p_* is the time for the preparation period, which includes water suppression and outer volume suppression; *τ*_1_ and *τ*_2_, are the durations of 90° and 180° pulses, respectively; *TE* is the spin echo time; *T_d _* is the time delay before data acquisition, which is used for slice selection in the *z*-direction and phase encoding in the *x*-*y* plane of the slice (not shown); *T*_acq  _ is the acquisition time; *T*_sec  _ is the time for one slice; the total repetition time *TR* for *N* slices is *TR* = *T*_sec_ × *N*_sec_.

The timing of the sequence is determined by hardware capacities and practical considerations. The times of *T_p_* and *T_d_*, as well as *τ*_1_ and *τ*_2_ for example, relate to the hardware and are optimized to the shortest possible times during the design of the pulse sequence. They are treated as constants in this application. *T*_acq_ and *N*_sec_ are variables selected by the users. *T*_acq_ equals *N*/SW, where *N* is the number of data points in the echo and SW is the spectral width, both of which are determined by practical considerations and compromises. For example, one way to reduce scan time, *T*_sec_, is to reduce the *TE*, which is possible by reducing *T*_acq_. Increasing spectral width can reduce *T*_acq_,but does so at the expense of increasing noise. Reducing *N* reduces *T*_acq_, but it will reduce the spectral resolution and produce truncation effects, as the acquisition may begin after the echo signal is fully built up and end before the echo signal is fully decayed, as shown in the dashed box in Figure 1. Although zero-padding the truncated echo prior to Fourier transformation may improve the spectral resolution, it will also produce wiggling in the spectrum. Both low digital resolution and wiggling will hinder spectral fitting in the frequency domain [15]. All these factors considered, SW and *N* were conventionally designated to be 1000 Hz and 256, respectively, for a 1.5T scanner [7] and 2000 Hz and 512 for 3T scanners. With these parameters, and for *PE* = 32 × 32 and *N*_sec_ = 4, the *TE* is 280 ms, yielding a *T*_sec_ of 575 ms, *TR* = 2300 ms, and a scan time of 30 minutes. Together with preparation time, including slice and outer volume suppression (OVS) band prescription, shimming, and prescanning, the scan time totals 50 min. Further increasing the number of slices will proportionally increase the scan time.

We reduced the number of data points in the echoes acquired on a 3T scanner from 512 to 256, so that 7 slices will be covered in a *TR* of 2300 ms. To avoid the disadvantage of low spectral resolution and the truncation effects on spectral fitting in the frequency domain associated with acquisition of a limited number of data points, we adopted an algorithm of spectral fitting in the time domain. Those details will be described below under *Data Processing section*.

### 2.2. Computer Simulation

We performed computer simulations to compare the effects of spectral fitting algorithms on signal truncation in the time domain and spectral resolution or sinc wiggles in the frequency domain. The signal simulated the 3 singlets of Ch, Cr, and NAA with amplitudes of 24, 36, and 48 (a.u.), respectively. The linewidth was 10 Hz for either a Lorentzian or Gaussian lineshape. For a Voigtian lineshape, the Lorentzian decay was 2.5 Hz, and the Gaussian decay was 7.5 Hz for all three lines. Spectral width was 2000 Hz, the number of data points in the echo was 256, and it was zero-filled to 512 or 1024. We also conducted a Monte Carlo simulation for signal fitting in the time domain and compared the standard deviations of the estimated amplitudes with their Cramer Rao Lower Bounds (CRLBs), a benchmark for assessing the accuracy of spectral fitting algorithms.

### 2.3. MR Data Acquisition

We carried out all MR measurements on a spectroscopic phantom and on 3 human volunteers, respectively, using a whole body 3T scanner (Signa HDx 3.0T, GE Healthcare, Waukesha, WI), equipped with a quadrature transmit/receive head coil, and an 8-channel receive-only head coil. First, we acquired scout images using a commercial gradient recalled echo-based 3-planar MRI sequence and then prescribed the localizer images of the MRSI slices, which were in axial plane for phantom scans and in an oblique axial plane parallel to the anterior commissure-posterior commissure line in human subjects. Then, we localized the MRSI slices by copying the location of the localizer images and acquired MRSI data using the multiplanar MRSI pulse sequence [7]. The number of slices, the slice thickness, and spacing varied in accord with those of different MRSI sessions (*vide infra*). However, typical parameters of the MRSI pulse sequence were as follows for both the phantom and human subjects: number of slices = 7; slice thickness = 10 mm; spacing between slices = 4 or 0 mm; nominal number of phase encodings (PEs) = 16 × 16 or 32 × 32; *TR*/*TE* = 2300/144 ms; spectral width = 2000 Hz; number of data points in the echo = 256. The duration of an MRSI scan was 8 or 30 minutes, depending on the number of PEs. Total scan time including MRI localizer, MRSI slice prescription, OVS band placement, and field shimming was about 26 or 50 minutes. When repeating the MRSI scans with different slice spacings, we changed only the spacing but not the position of the central (4th) slice. Therefore, we used signals from this slice to assess the effects of differing slice spacings on the “crosstalk” between slices. For each MRSI scan with a differing spacing between slices, we performed an autoprescan for field shimming and transmitter gain optimization. Then receiver gains (RGs) were manually adjusted if needed to retain the same RG values for all MRSI scans. The protocol was approved by the Institutional Review Board of the New York State Psychiatric Institute. Written informed consent was obtained from each human participant.

### 2.4. Data Processing

#### 2.4.1. Combination of Multichannel Data

The *k*-space MRSI data from individual coil elements were transformed to the image domain using a 2D spatial Fourier transform after spatial filtering that used a Hamming window function. The data from the multiple coil elements were combined using the following procedures.

**(1) Water Signal Removal**

We used a matrix-pencil-method-based procedure [16] to decompose the signal, identify water components by their frequencies, and remove them from the signal [16]. This method was able to remove water signal almost completely (>98%) without interfering with the metabolite signals of interest.

**(2) Removal of Corrupted Points**

We replaced the first 6 echo data points [17], which were corrupted by the activation of the analogue-to-digit converter, with 6 extrapolated points derived from signal parameters that were estimated from the uncorrupted data points using the matrix pencil method.

**(3) Data Apodization**

We next multiplied the cleaned echo data by a Gaussian function, *G*(*t*) = *e* ^−*βt*^2^^, to suppress noise and reduce baseline distortion, albeit at the expense of line broadening. The line broadening was 10 Hz for phantom data and 4 Hz for *in vivo* data.

**(4) Phase Alignment**

We eliminated voxelwise phase differences in echoes from individual coil elements by subtracting the phases at the top of their echoes.

**(5) Weighted Summation**

We summed the phase-aligned echoes using weighting factors that were proportional to the echo amplitudes and inversely proportional to the noise levels of the coil elements. Noise levels were determined by measuring the standard deviations of the data points in the signal-free regions of the frequency domain spectra of a phantom.

#### 2.4.2. Spectral Fitting

We quantified the spectral components using the following general model function to fit the echoes:

(1) $$S\left(t\right)=\overset{M}{\underset{m=1}{\sum }}A_{m}e^{i(2\pi f_{m}t+\varphi_{m})}e^{-\alpha_{m}|t|-\beta_{m}t^{2}},$$

where *A_m_*, *f_m_*, **φ*_m_*, and **α*_m_* represent the amplitude, frequency, phase, and Lorentzian decay of peak* m*, respectively;  **β*_m_* is the Gaussian decay. Note that the *t* runs from −dt · *N*/2 to dt · (*N*/2 − 1), where dt is dwell time and *N* is the number of data points in the echo. Note also that when fitting the spectrum with a pure Lorentzian model, we set **β*_m_* to be zeros; when fitting the spectrum with pure Gaussian model, we set the **α*_m_* to be zeros; when fitting the spectrum with a Voigtian model, we set the **β*_m_* to be the same for all *M* peaks.

Further notes are warranted for the process of spectral fitting. The signal parameters in (1) were determined using a nonlinear least squares fitting routine in Matlab© (2007a, The MathWorks, Natick, Massachusetts). To ensure that these parameters were real numbers, the real parts and imaginary parts of both the model function in (1) and the measured echo signal were concatenated, respectively, to form real-number series. It is important to determine accurately the initial values for *A_m_*, *f_m_*, and **α*_m_*, as well as the number of peaks, to ensure the robustness of the fitting. They were estimated from the magnitude spectrum obtained by zero-padding the echo to 4096 points and then performing FFT, whereas the global initial phase was obtained by the phase of the top point of the echo.

#### 2.4.3. Comparing Data Acquired with or without Slice Spacing

The influence of acquiring contiguous slices on signal intensity was determined by comparing signal amplitudes from the two scans with or without spacing between slices. We selected voxels from a region within the brain in the 4th slice whose location was the same for the scans with or without spacing. The amplitudes of the *m*-th signal components (peak areas in the frequency domain), *A_m_*, were used to calculate the relative differences of the signals obtained with or without spacing

(2) $$d_{m}=\frac{\left(A_{m,w\cdot \text{sp}}-A_{m,\text{wo}\cdot \text{sp}}\right)}{A_{m,w\cdot \text{sp}}}.$$

We used the means and standard deviations (S.D.) of the *d'*s to evaluate the reductions in signal caused by the “crosstalk” introduced between slices when spacing was removed.

## 3. Results

### 3.1. Comparing Signals with and without Spacing between Slices

Tables 1 and 2 show the relative differences of the amplitudes, calculated using (2), of the phantom and *in vivo* MRS signals acquired with and without spacing between slices. The phantom data did not show significant differences in these values (Table 1). The mean values of the differences in amplitudes (or the signal reduction) caused by the “crosstalk” in the *in vivo* data was <7.5% for the first two tests, when *TR* = 2.3 s. On the average, the signal reduction was smaller for the third test when *TR* = 3.0 s (Table 2). 

### 3.2. Comparison of Spectral Fitting in the Time and Frequency Domains

**Figure 2:**
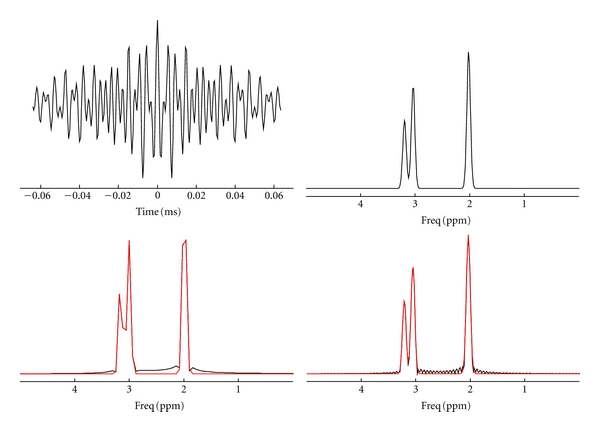
Simulated echo with 256 data points and its spectrum obtained with 1024 data points (upper). The time and frequency domain fitting algorithms can fully recover each of them. However, when fitting the spectrum with 256 data points, or when the spectrum was zero-filled from 256 to 1024 data points, sizeable errors are evident as a consequence of either low spectral resolution or truncation effects, which manifest as wiggles in the spectrum after zero-filling (lower).

Errors can be as large as 4% when fitting the noise-free original data in the frequency domain with a Lorentzian or Gaussian lineshape, or up to 15% for fitting data with a Voigtian lineshape (Table 3). These errors are the consequence of low spectral resolution and a (Voigtian) model mismatch in the frequency domain. When fitting the zero-padded signals (from the original 256 to 1024 points) in the frequency domain, errors were of the same order, whereas errors were reduced to zero when fitting the spectrum with the original 1024 points. This finding indicates that errors using padded signals are caused by wiggles (Figure 2). In contrast, when fitting the original 256 points of noise-free signals in the time domain, the signals can be perfectly recovered, regardless of whether the line shapes are Lorentzian, Gaussian, or Voigtian. Monte Carlo simulations with 400 noise realizations added to the 256 points time domain signals revealed that the estimated amplitudes of NAA, Cr, and Ch approximated the true values for all three lineshapes. The SDs of the Lorentzian and Voigtian lineshapes were less than 2 times the Cramér-Rao Lower Bounds (CRLBs), whereas the SDs of the Gaussian line shapes were approximately 1.5 times the CRLB (Table 4).

### 3.3. Combination of Multichannel Signals

**Figure 3:**
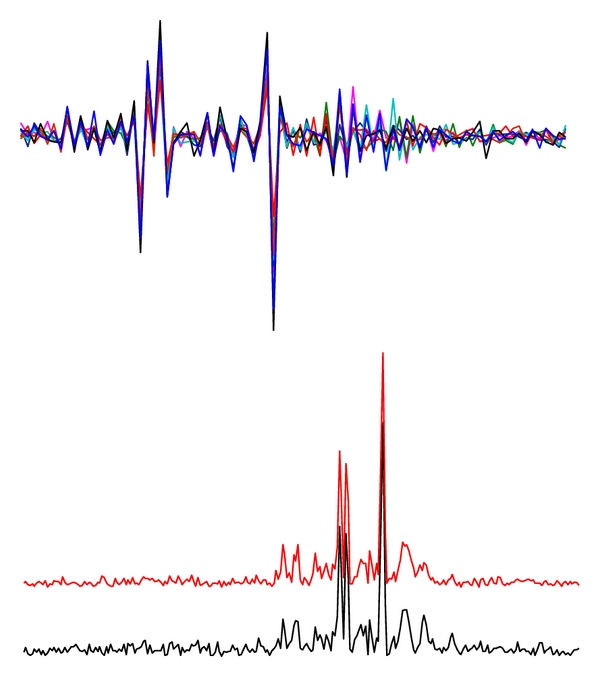
Examples of the combination of *in vivo* signals from a multichannel coil array. Upper: phase alignment displays the real parts of the spectra without phase correction. Lower: a combined spectrum (red) and a channel spectrum with the highest SNR, shown in absolute mode. The SNRs of the combined spectrum and the channel spectrum with the highest SNR were 77.8 and 46.1, respectively.

The phase differences caused by the differing positions of coil elements were eliminated, yielding perfect alignment of the array signals in the frequency domain and thereby enhancing SNR (Figure 3).

### 3.4. Spectral Fitting and Metabolite Maps

**Figure 4:**
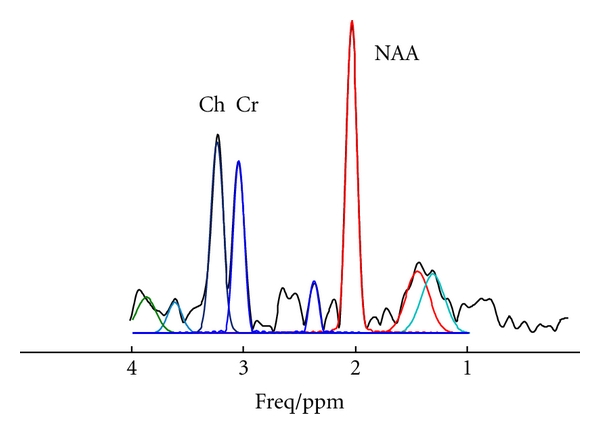
An example of spectral fitting of the *in vivo *data in the time domain for 256 data points, displayed in the frequency domain and absolute mode. Shown in colors are the individual fitted spectral lines overlaid on the measured spectrum in black. The excellent fitting supports the merit of spectral fitting in the time domain.

**Figure 5:**
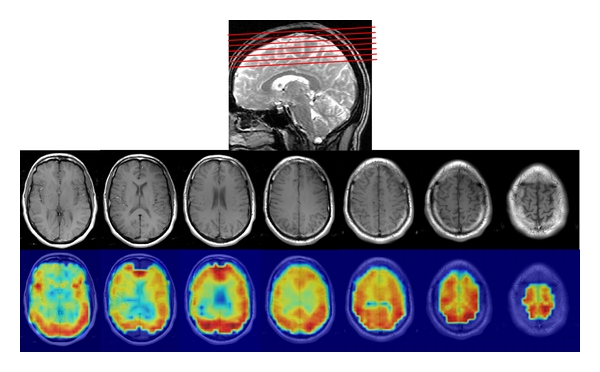
Slice prescription (top), localizer images (middle), and NAA images overlaid on localizers (bottom). The slices are contiguous and cover a large volume of the brain.

The performance of spectral fitting of the data of 256 points in the time domain is further demonstrated using *in vivo* data. An example of the spectral fitting is shown in Figure 4, in the form of an absolute spectrum. The whole brain MRSI of NAA is shown in Figure 5, overlaid on their localizer images. Similar results were obtained for the other two subjects.

## 4. Discussion

We reported herein improvements of the well-known and widely used multi-section MRSI technique. The modified pulse sequence has several important advantages over its standard implementation: (1) it eliminates spacing between slices; (2) it allows increase in the number of slices (in our study from 4 to 7) without the expense of increasing scan time; (3) it permits a reduction in slice thickness and therefore improves spatial resolution, which can be achieved without the expense of reducing overall volume coverage because of the increased number of slices that are acquired; (4) it employs a multichannel coil for data acquisition to improve SNR. It also employs fitting of the severely truncated full echo in the time domain. These improvements make the multi-section MRSI technique more diagnostically valuable.

Compared with the performance of the original MRSI pulse sequence, each modification individually can have its own unique advantages, limitations, and challenges. Spacing between slices, for example, was introduced in the original implementation of the sequence to avoid “crosstalk” between adjacent slices, a phenomenon of signal interference caused by the imperfect slice profiles in which edges are not clearcut but instead interlace with one another. “Crosstalk” can cause signal loss and thus reduce SNR. Slice spacing, however, comes at the expense of information loss from the volumes between slices that are not imaged, which can be as large as 30% of the total MRS imaging volume. This reduced volume of coverage also reduces overall spatial resolution. The reduction in signal caused by eliminating spacing between slices was less than 10% *in vivo* using our modified MRSI sequence (Table 2). This loss of signal would be regarded as substantial if the SNR of the original signal was low, but it is in fact an inconsequential loss when using an 8-channel multichannel coil array, which typically doubles SNR compared with use of a standard quadrature coil [8, 11, 12]. Therefore, even with a 10% signal reduction caused by “crosstalk” from contiguous slices, the SNR using a multichannel coil in conjunction with our modified sequence is still much higher than the SNR when using a standard quadrature head coil without “crosstalk” (Figure 4). We note, in fact, that increasing the number of slices from 4 to 7 can actually help to reduce the “crosstalk” across slices, because interleaved slice excitations are separated temporally by at most one interval if the number of slices is 4 (i.e., 1-3-2-4), whereas with 7 slices, the temporal interleave can be 2 or 3 intervals for adjacent slices (e.g., 1-3-5-7-2-4-6, where slices 1 and 2 are temporally separated by 3 intervals, slices 4 and 5 are separated by 2 intervals, and so on). In addition, the reduction in slice thickness theoretically would lead directly to a proportional reduction in SNR when shimming is perfect. In reality, however, and especially in regions near air-tissue interfaces, the effect of reduced volumes in each slice on SNR is not linear because smaller volumes come with narrower line widths. Consequently, we did not observe significant signal drop-out in regions of air-tissue interface within the lower frontal lobe (Figure 5). Thus, the advantages that our modified multi-section MRSI sequence provides are the whole brain coverage with contiguous slices, potentially improved spatial resolution, and improved spectral line width.

The reduction in number of data points in the echo, which is the core of the current modification, poses severe challenges for spectral fitting and spectral quantification. Computer simulation (Table 3) showed that fitting a spectrum with low spectral resolution in the frequency domain can produce sizeable errors. Conversely, increasing the spectral resolution can improve the accuracy of spectral fitting in the frequency domain. When the number of data points in the original echo was 1024, the frequency domain fitting algorithm perfectly recovered the spectrum. However, significant errors remained if the spectrum was obtained by zero-filling the 256 data points to 1024 (Table 3), reflecting the detrimental effects of truncation or wiggle artifacts on spectral fitting. Spectral fitting in the time domain, in contrast, accurately fits the echo using 256 data points, suggesting that time domain spectral fitting is preferable for signals with fewer data points, a possibility that Monte Carlo simulation of the signal (Table 4) and spectral fitting of the *in vivo* data (Figures 4 and 5) are verified.

Our implementation of whole brain metabolite mapping using a multiple 2D MRSI sequence also affords distinct advantages over 3D MRSI, in which the number of phase encoding (PE) steps in the 3rd dimension is typically 8 [18, 19]. This small number of data points entails pronounced, long-range signal contamination across slices because of the effects of the point spread function when reconstructing the slice data directly using FFT. Spatial filtering with Hamming, Hanning, or Kaiser window functions must be applied prior to FFT to suppress this signal contamination, but at the expense of increasing the amount of signal bleeding between adjacent slices. Therefore, the effective slice thickness of 3D MRSI is approximately 1.4 times that of the nominal slice thickness, which significantly degrades spatial resolution. The effective thickness of slices using multiple 2D MRSI, on the other hand, is close to its nominal value, despite the fact that the slice profile is not ideal and slight “crosstalk” is present between slices. Another limitation of 3D MRSI using PRESS localization is that the first and last slices cannot be used, reducing the effective number of slices by 2 [18, 19]. Our pulse sequence, in contrast, provides high-quality MRSI images in all 7 slices (Figure 5). 3D MRSI has a distinct advantage over multi-section MRSI, however, in that the location of slices in 3D MRSI can be shifted to specific anatomical regions of interest by employing a phase shift of the Fourier transform.

Comparing signal losses in phantom and *in vivo *MRSI data in the absence of spacing between slices showed that signal loss caused by “crosstalk” is related to the slice profile, the spatial and temporal separations between successive excitations, and the longitudinal relaxation times of the subjects. Our experimental procedures ensured that the spatial and temporal separations of excitations were identical for the phantom and *in vivo* scans. The longitudinal relaxation times of molecules in the phantom were significantly longer than those in the human brain, and therefore comparatively larger signal loss could be expected if all other conditions remained the same. The fact that signal loss was negligible in the phantom (Table 1) but was approximately 10% in the human brain (Table 2) when spacing was eliminated as compared with 4 mm spacing can be attributed to a better slice profile in the phantom. Comparing spectral fitting in the time domain with fitting in the frequency domain has been of long-standing interest in MRS [20–25]. As the time domain and frequency domain signals are related by the Fourier transform, they theoretically have the same information content. However, fitting in the time and frequency domains may have their unique advantages and disadvantages depending on the properties of the signal, such as noise level and phase or baseline distortions [20–25]. Time domain methods are preferable in the presence of distortions in the measured signals, including truncation [20, 23]. Our computer simulation provided a numerical example for the detrimental effects of truncation in the frequency domain and showed that fitting in the time domain is immune to those effects.

In conclusion, we have presented a realization of extended brain metabolite mapping using a multiple 2D MRSI pulse sequence in conjunction with use of a multichannel RF coil and spectral fitting in the time domain. These combined techniques have permitted an increase in the number of slices from 4 to 7, without sacrificing scan time or SNR. The extended brain coverage, reduced slice thickness, and increased SNR can potentially make the sequence more clinically valuable.

##  Grant Support 

This study was funded in part by NIMH grants K02 74677, MH089582, MH068318, 1P50MH090966, NIEHS grant ES015579, and NIDA grants DA027100, and DA017820.

## Figures and Tables

**Table 1:** Comparisons of signal amplitudes acquired on a phantom with or without spacing between slices. Means and SD were calculated using (2). *n* is the number of selected voxels in the 4th slice. The *TR* was 2.3 s for tests 1 and 2, and 3.0 s for test 3. The labeled concentrations for NAA, Cr, and Cho were 12.5, 10.0, and 3.0 mM, respectively.

Test	NAA (mean ± SD)	Cr (mean ± SD)	Cho (mean ± SD)
1 (*n* = 47)	1.26% ± 4.16%	1.71% ± 3.23%	1.05% ± 5.82%
2 (*n* = 39)	0.51% ± 4.22%	0.07% ± 6.15%	−0.47% ± 4.17%
3 (*n* = 49)	0.47% ± 3.29%	−1.73% ± 4.43%	−0.84% ± 4.82%

**Table 2:** Comparisons of signal amplitudes acquired on a human subject with or without spacing between slices. Means and SD were calculated using (2). *n* is the number of selected voxels in the 4th slice. The *TR* was 2.3 s for tests 1 and 2, and 3.0 s for test 3.

Test	NAA (mean ± SD)	Cr (mean ± SD)	Cho (mean ± SD)
1 (*n* = 34)	2.50% ± 4.47%	7.30% ± 4.09%	2.94% ± 9.71%
2 (*n* = 35)	7.33% ± 4.24%	1.56% ± 6.23%	5.63% ± 6.64%
3 (*n* = 38)	2.80% ± 5.95%	3.04% ± 8.07%	2.52% ± 10.0%

**Table 3:** Relative errors (%) of spectral fitting of the simulated signal in the frequency domain, caused by truncation effects and by wiggles, respectively.

Lineshape	Original 256 points			Zero-padding to 1024 points		
	NAA	Cr	Ch	NAA	Cr	Ch
Lorentzian	4.72	2.04	4.34	4.29	3.23	0.82
Gaussian	4.06	0.19	1.72	4.38	2.10	0.44
Voigtian	8.45	15.31	6.07	7.00	9.33	0.59

**Table 4:** Results of Monte Carlo study of the estimated amplitudes (mean ± SD). The true values of NAA, Cr, and Ch are 48, 36, and 24, respectively; the CRLBs are 0.1574, 0.1288, and 0.1858 for Lorentzian, Gaussian, and Voigtian, respectively.

Lineshape	NAA	Cr	Ch
Lorentzian	47.99 ± 0.27	35.99 ± 0.31	24.02 ± 0.30
Gaussian	48.00 ± 0.18	36.00 ± 0.19	24.00 ± 0.19
Voigtian	48.00 ± 0.37	36.00 ± 0.40	24.00 ± 0.38
